# Abnormal development of corticospinal tracts in children with Tourette syndrome: A single-center retrospective study

**DOI:** 10.1097/MD.0000000000044702

**Published:** 2025-10-10

**Authors:** Hong-Xin Jiang, Yan-Mei Ju, Ting-Ting Gao, Jian-Li Sun, Hong-Gang Wang, Lei Cao, Shu-Man Han, Hui-Zhao Wu, Jin-Xu Wen, Bu-Lang Gao, Wen-Juan Wu

**Affiliations:** a Department of Radiology, Hebei Provincial Gucheng County People’s Hospital, Gucheng, China; b Department of Radiology, Hebei Medical University Third Hospital, Shijiazhuang, China.

**Keywords:** corpus callosum compression part, corticospinal tract, diffusion tensor imaging, magnetic resonance imaging, Tourette syndrome

## Abstract

To explore the abnormal changes and localization of never fiber bundles on magnetic resonance imaging diffusion tensor imaging (DTI) in children with Tourette syndrome, children with Tourette syndrome were retrospectively enrolled to undergo three-dimensional (3D) T1 + DTI sequence scanning, raw data collection of 1 to 15 year old head, and cross-sectional spacing of 0.5 mm on DTI scanning. The specific software was used to display relevant nerve fiber bundles on DTI. In total, 7 children with Tourette syndrome were enrolled including 6 boys and 1 girl aged 3 to 10 (5.6 ± 2.3) years. The symptoms included paroxysmal shoulder convulsions, facial muscle twitching, involuntary blinking, right hand twitching, epilepsy, involuntary shrug, and attention deficit. On DTI, the morphology and course of the corticospinal tracts were damaged or abnormal, the inferior frontal occipital tracts were damaged or abnormal, the fiber bundles in the compression part of the corpus callosum were reduced in the number and abnormal in the morphology, and the brain nerve fiber bundle connection was reduced. In conclusion, the nerve fiber bundle connection and damage and abnormality of the morphology and course of the corticospinal tract, fiber bundles of the compression part of the corpus callosum, and the inferior frontal occipital tract in children with the Tourette syndrome can be clearly observed on DTI, and the DTI technique can provide valuable imaging support for revealing the neuropathological mechanism of the Tourette syndrome in children.

## 1. Introduction

Tourette syndrome is also called multiple tics-coprolalia syndrome or Gilles de la Tourette syndrome, which is a disease of neuropsychiatry in childhood and adolescence. The major manifestation of this syndrome is a rapid, aimless, repetitive, and involuntary motor tic in some parts of the body, frequently accompanied by depression, sleep disorder, emotional and obsessive–compulsive disorder, and attention deficit hyperactivity disorder.^[1–4]^ Tourette syndrome is not a rare disorder, with an incidence ranging 1% to 2% in children of school ages.^[1,5,6]^ Boys have a clearly higher prevalence, with a boy to girl ratio of 3:1 to 4:1. It often presents as a benign process, but there are also patients who are difficult to treat. Generally, it can self heal within a short period of time or be cured through treatment. For refractory patients, it may take several years to be healed and even continue into adulthood. Currently, its pathogenesis is unknown. Studies have found some abnormalities in the frontal lobe–corpus striatum–thalamic loop as the primary pathology of the Tourette syndrome, but the evidence in medical imaging supporting this conclusion is inconsistent.^[4,7]^ Diffusion tensor imaging (DTI) of the magnetic resonance imaging (MRI) can help observing the connectivity and integrity of the live tissue structure, displaying the direction of nerve fiber bundles in the white matter, and conducting fine medical imaging of the central nerve fibers.^[4,8]^ The DTI has been used in inspecting the cerebral structure as well as function of the brain in children with Tourette syndrome, but without consistent conclusions. Abnormal DTI-parameter changes have been reported in the basal ganglia, thalamus, caudate nucleus, putamen, prefrontal area, and cingulate gyrus.^[4,9,10]^ Currently, no studies have reported on the damage and abnormality of the morphology and course of the nerve fiber bundles of the corticospinal tract, fiber bundles of the compression part of the corpus callosum, and the inferior frontal occipital tract in children with the Tourette syndrome together with the nerve fiber connection in the brain. This study was thus performed to investigate the nerve fiber connection and possible damage and abnormality of the morphology and course of the above fiber bundles in Tourette syndrome using the DTI technique.

## 2. Materials and methods

### 2.1. Subjects

This retrospective single-center study did not need an approval from the ethics committee of Hebei Provincial Gucheng County Hospital, and informed consent was obtained from all patients for participating in and publication of the clinical data. Children with Tourette syndrome were retrospectively enrolled between March 2021 and March 2024. The Helsinki Declaration has been followed for involving human subjects in the study. The inclusion criteria were children aged 1 to 15 years old with Tourette syndrome who had not been treated prior to the MRI examination or who did not have any contraindications for MRI scans. The exclusion criteria were children with other organic diseases in the brain, such as Huntington disease, chorea minor, Wilson disease, and sequelae of encephalitis, without any medication that could cause movement disorders before becoming ill, or without bad image quality caused by head movement artifacts during the process of examination. The data collected were deidentified.

### 2.2. DTI examination equipment and technical parameters

The PHILIPS Achieva 3.0 T conventional MRI scanner was used for the examination, and no abnormal signals were found in intracranial areas before DTI scanning. DTI scan was performed after the child’s head was fixed with a sponge pad to guard against head movement. The child remained conscious and used cotton balls to block both external ear canals. The three-dimensional (3D) T1 + MRI DTI sequence scanning was selected with a cross-sectional spacing of 0.5 mm and software to display information on the position of relevant nerve fiber bundles. In the scanning of MRI DTI, the parameters were: sequence of spin echo planar imaging (SE-EPI), axial scans, TR (repetition time) = 6100 ms, TE (echo time) = 87 ms, layer thickness = 2.5 mm, interval of 0, 50 layers totally, intralayer resolution = 128 × 128, FOV (field of view) = 256 mm × 256 mm, 64 diffusion gradient directions, b = 1000 s/mm^2^, 8 b = 0 images, and NEX = 2. The parameters of structural MRI scanning were: 176 slices, repetition time = 1900 ms, echo time = 3.44 ms, flip angle = 9°, slice thickness = 1 mm, inversion time = 900 ms, FOV = 256 mm × 256 mm, and acquisition matrix = 256 × 256. The parameters of structural MRI (sMRI) scanning were as follows: sagittal scan, TE 3.39 ms, TR 2530 ms, 128 layers, layer thickness 1.33 mm, interval of 0 mm, TI 1100 ms, FOV 256 mm × 256 mm, in-plane resolution 256 × 192, and FA 7 degrees.

### 2.3. Image postprocessing of nerve fiber bundles

The scanned DTI raw data were transferred to the DTI Studio software on the MR WorkSpace workstation for smoothing and denoising before generation of the fractional anisotropy (FA) and apparent diffusion coefficient (ADC) maps. The continuous tracking (FACT) algorithm was for fiber assignment in tracking fiber bundles around the nucleus accumbens with the threshold setting: FA < 0.25. After confluence of the obtained DTI image with the 3DT1 sequence, the fiber bundles were reconstructed and the positional relationship was identified between the nucleus accumbens and the corresponding nerve fiber bundle.

### 2.4. Statistical analysis

The SPSS software 20.0 (IBM, Chicago) was used for the statistical analysis of this study. Measurement data were expressed as mean and standard deviation if in the normal distribution. Categorical data were demonstrated as frequency and percentage.

## 3. Results

Seven children with Tourette syndrome were enrolled including 6 boys and 1 girl aged 3 to 10 (5.6 ± 2.3) years. The symptoms included Paroxysmal shoulder convulsions, facial muscle twitching, involuntary blinking, right hand twitching, epilepsy, involuntary shrug, and attention deficit (Table 1).

**Table 1:** Demography, symptoms and nerve fiber bundle damage in patients with Tourette syndrome.

Cases	Sex	Age	Symptoms	Corticospinal tract damage	Corpus callosum compression part	Inferior frontal occipital tract	Reduced nerve connectivity
1	M	3	Paroxysmal shoulder convulsion	Yes	No	No	No
2	M	4	Facial muscle twitching and involuntary blinking	Yes	Yes	No	Yes
3	M	10	Right hand twitching	Yes	No	No	No
4	M	5	Tourette syndrome	No	Yes	Yes	No
5	F	6	Epilepsy	Yes	No	No	No
6	M	7	Involuntary shrug	Yes	No	No	No
7	M	4	Attention deficit and Tourette syndrome	Yes	Yes	Yes	Yes

**Figure 1.**
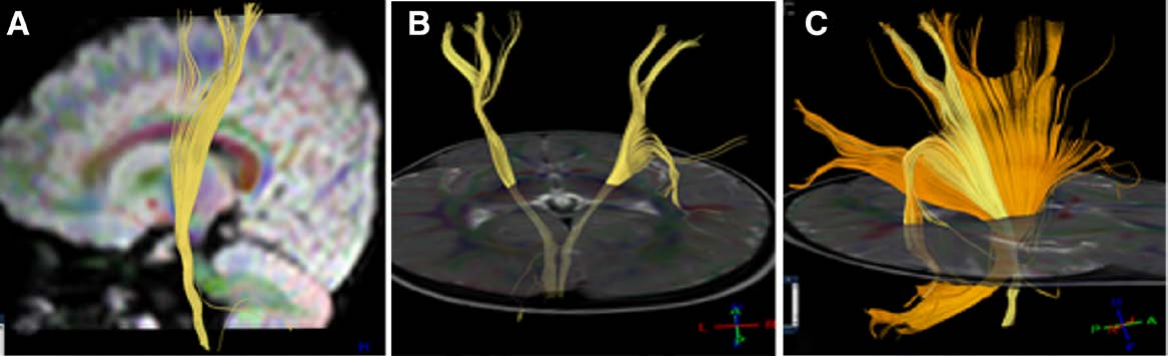
Corticospinal tract and cortical cerebellar tree in case 1. (A) Left side view: the morphology and course of the left corticospinal tract were normal. (B) Back view: some fibers in the right corticospinal tract did not reach the cortex and bended towards the temporal lobe in the radiating coronal region. (C) Right side view: the yellow corticospinal tract on the right side and the brown cortical cerebellar tree on the right side both had some fibers reaching the temporal lobe cortex.

**Figure 2.**
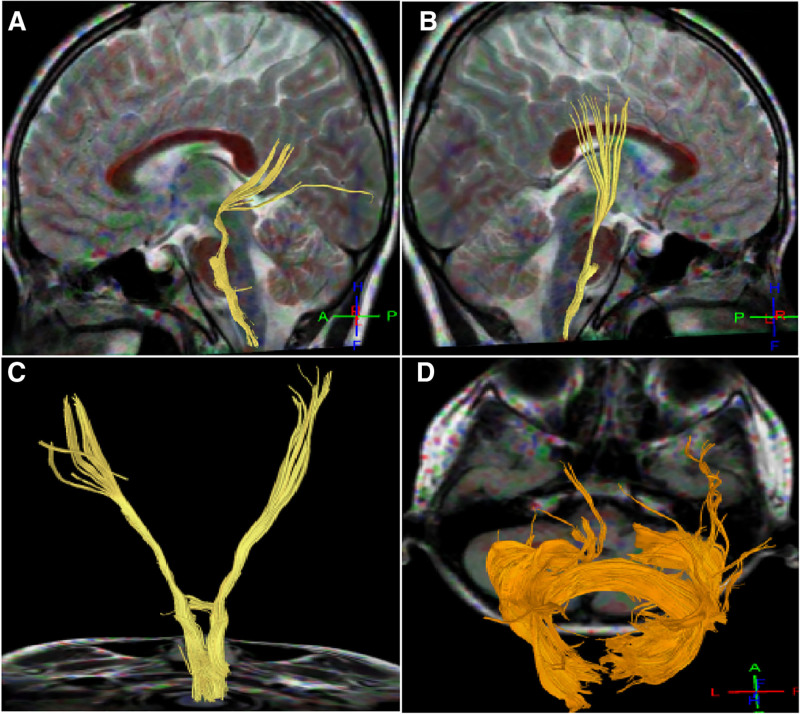
Corticospinal tracts and corpus callosum in case 2. (A–C) Abnormal development of both corticospinal tracts was present, with most fiber bundles not reaching the functional area of the dermis layer on the left side view (A), right side view (B), and back view (C). (D) Top view: sparse fiber bundles in the tapetum of the corpus callosum were present on both sides with reduced brain network connectivity.

**Figure 3.**
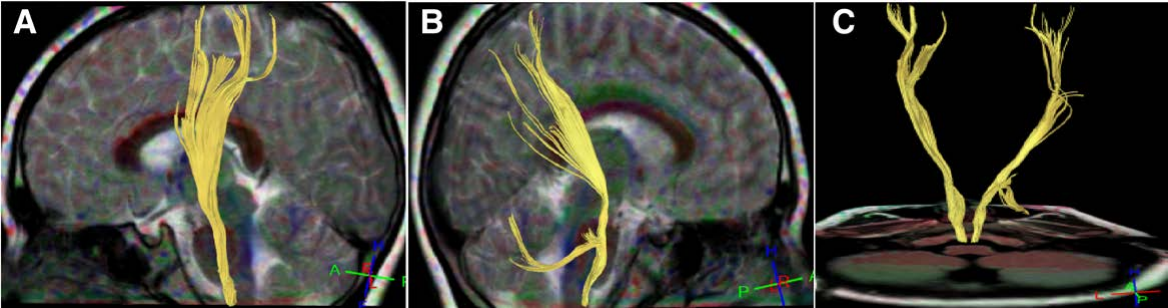
Corticospinal tracts and cerebellar hemisphere in case 3. (A) Left side view: a small amount of fiber bundles in the left corticospinal tract did not reach the cortex. (B) Right side view: abnormal fiber bundles in the right corticospinal tract at the pons reached the cerebellum with a small amount of fiber interruption. (C) Back view: of the bilateral corticospinal tracts, some fiber bundles on the right side ran to the cerebellar hemisphere.

**Figure 4.**
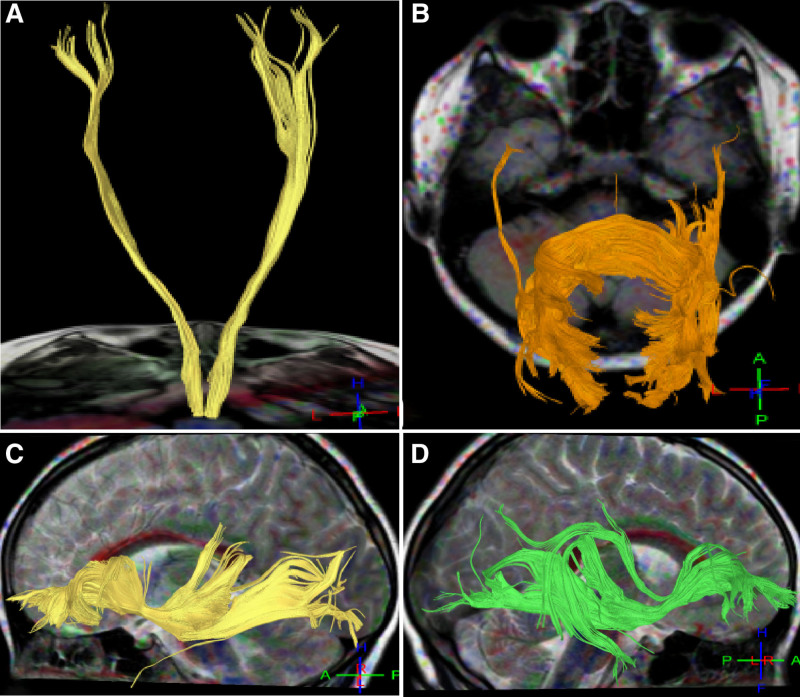
Corticospinal tracts, inferior frontal occipital fasciculus and the parietal occipital cortex in case 4. (A) Back view: the yellow corticospinal tract on both sides were normal in the morphology and course. (B) The fiber bundles in the compression part of the corpus callosum had significant decreases in brain network connectivity, especially in the tapetum of the corpus callosum. (C and D) The inferior frontal occipital fasciculus on both sides had a small amount of fibers which did not reach the parietal occipital cortex.

**Figure 5.**
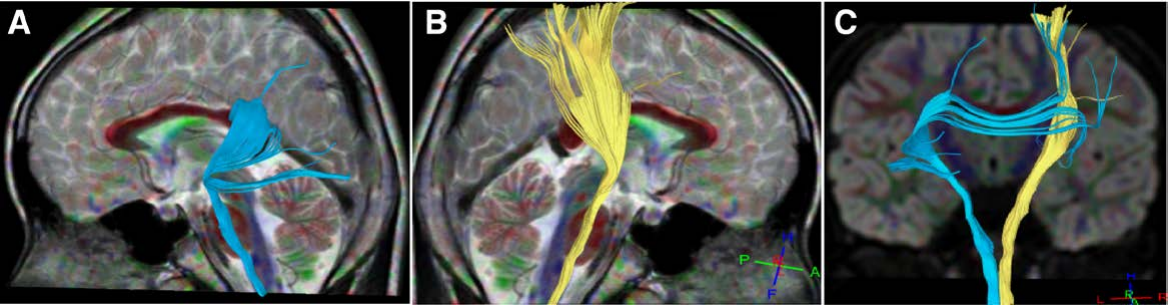
Corticospinal tree, corpus callosum, and motor cortex in case 5. (A) Left side view: the left corticospinal tree (cyan) had abnormal morphology and courses and did not reach the cortex, with some fiber bundles crossing the midline through the corpus callosum to reach the contralateral cerebral hemisphere. (B) Right side view: the corticospinal tract (yellow) on the right side had no abnormalities observed. (C) Back view: part of the fibers in the left corticospinal tree accompanied the right corticospinal tree to reach the contralateral motor cortex area, and some terminated in the internal capsule area.

**Figure 6.**
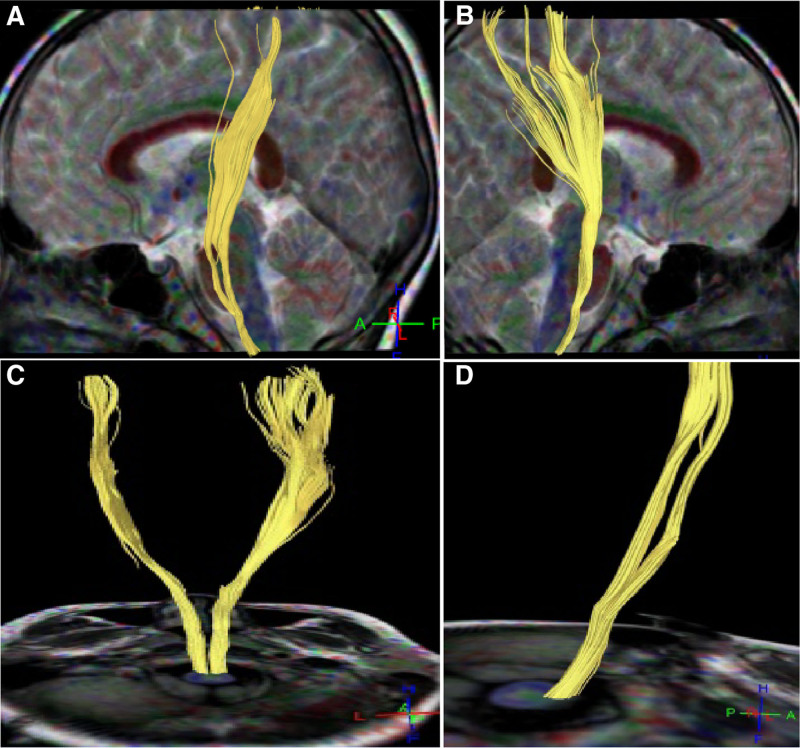
The corticospinal tract in case 6. (A) Left side view: some of fiber bundles of the left corticospinal tract were interrupted in the pons area. (B) Right side view: the corticospinal tract on the right side was normal. (C and D) Back view: of bilateral corticospinal tracts, a clear defect area was present in the left corticospinal tract in the brainstem area in the enlarged view (D).

**Figure 7.**
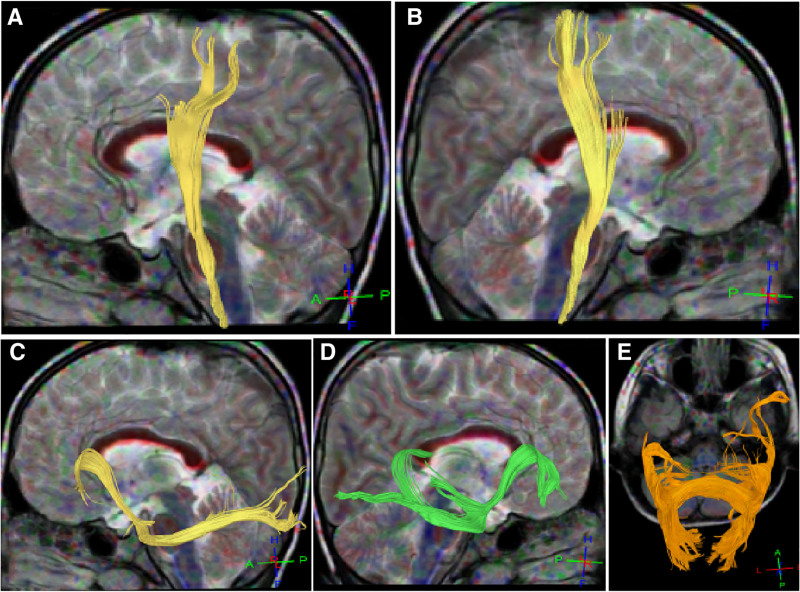
Bilateral corticospinal tracts, bilateral inferior frontal occipital fasciculus, and corpus callosum. A&B. Left (A) and right (B) side view: A small amount of fiber bundles of the bilateral corticospinal tracts did not reach the cortex. C&D. Left (C) and right (D) side view: the morphology and course of the bilateral inferior frontal occipital fasciculus were abnormal, with most fiber bundles not reaching the cortex. (E) The fibrous bundles in the compression part of the corpus callosum had abnormal morphology and reduced brain network connectivity.

On DTI, the morphology and course of the corticospinal tracts were damaged or abnormal in all 7 (100%) patients (Table 1 and Figs. 1–7), the fiber bundles in the compression part of the corpus callosum were reduced in the number and abnormal in the morphology in 3/7 (42.86%) cases (Figs. 2, 4, and 7), the inferior frontal occipital tracts were damaged or abnormal in 2/7 (28.57%) patients (Figs. 4 and 7), and the brain nerve fiber bundle connection was reduced in 2/7 (28.57%) patients (Figs. 2 and 7).

## 4. Discussion

This study investigated the changes and localization of never fiber bundles on MRI DTI in children with Tourette syndrome and it was found that the morphology and course of the corticospinal tracts were damaged or abnormal, the inferior frontal occipital tracts were damaged or abnormal, the fiber bundles in the compression part of the corpus callosum were reduced in the number and abnormal in the morphology, and the brain nerve fiber bundle connection was reduced. The corticospinal tract, fiber bundles of the compression part of the corpus callosum, and the inferior frontal occipital tract in children with the Tourette syndrome could be clearly observed on DTI, and the DTI technique could provide valuable imaging support for revealing the neuropathological mechanism of the Tourette syndrome in children.

The Tourette syndrome in children often occurs in boys aged 5 to 10 years as a sudden, brief, repetitive, or stereotyped twitching of 1 or 2 groups of muscles.^[11–13]^ Manifestations of this syndrome include blinking, frowning, showing teeth, making strange looks, shrugging, neck turning, nodding, body twisting, arm shaking or kicking, lower limb twitching, etc. These symptoms can be intensified by emotional tension and weakened by mental concentration and may disappear during sleep. At a certain period, the Tourette syndrome may be presented as a group of muscles twitching as the main symptom, however, at another period, it may manifest as another group of muscles twitching, which is the variability of symptoms. The course of the disease lasts for several months to a year. The frequency and severity of tics vary, with mild cases having no impact on the learning and living of the child, while severe cases affecting learning and disrupting the environment with the child even being unable to attend classes. The clinical manifestations of the Tourette syndrome are chronic fluctuation and multiple tics, accompanied by abnormal behaviors such as hyperactivity and compulsion. Currently, the etiology and pathogenesis of the Tourette syndrome in children are not fully understood in clinical practice, and it is mainly believed to be related to genetic, psychological and environmental factors, and neurotransmitter imbalance in children.^[14–16]^

The rapid development of the medical image processing technique has laid a solid theoretical foundation for the implementation of medical clinical diagnosis and treatment tools. Especially in neuroscience, MRI plays a particularly important role in brain structure recognition and medical image quantification analysis.^[4,17,18]^ It can assist in surgical planning and postoperative evaluation, detect brain abnormalities, draw brain function maps, investigate brain development, and conduct neuroanatomical analysis. It has become an important research field in medical image analysis and processing. By summarizing the correlation among abnormal development of the corticospinal tract, corpus callosum compression area, and inferior frontal occipital tract in children with Tourette syndrome, we can open up a new approach for functional research in children with Tourette syndrome. The white matter nerve fiber bundles in the brain and the functional area of the cerebral cortex are of equally important functional significance. Improving our understanding and comprehension of the white matter fiber bundles not only helps to explore the cognitive function mechanism of the brain, but also has important significance for clinical diagnosis and treatment. DTI is the process of applying diffusion sensitive gradients in multiple directions and calculating the feature vector values of the main diffusion direction within each voxel of brain tissue.^[4,17,18]^ After postprocessing, it obtains FA maps, color tensor maps, and white matter fiber bundle maps that reflect the diffusion features of cerebral white matter structure, and it was found that developmental abnormalities in the corpus callosum, corticospinal tract, and inferior frontal occipital tract are highly correlated with and account for the Tourette syndrome in children.

DTI is an extremely useful tool in investigating abnormalities of the brain white matter, whereas FA is widely applied to measure the extent of directionality of cellular structures in the fiber tracts which may reflect the cerebral microstructural integrity.^[1]^ Widespread white-matter abnormalities in patients with the Tourette syndrome have been demonstrated by some DTI studies, however, the results are inconclusive. Some studies have suggested decreased FA values in the corpus callosum,^[10,19]^ both superior longitudinal fascicles,^[20]^ both frontal lobes,^[10,20]^ and left internal or external capsule region,^[10,19]^ whereas others found elevated FA in the left postcentral gyrus^[21]^ or no significant FA differences between Tourette syndrome patients and normal controls.^[22–25]^ This inconsistency may be caused by the cohort size, cohort heterogeneity, and/or differences in the methodology and needs further investigation. After investigating the corpus callosum of boys with “pure” Tourette syndrome without treatment,^[26]^ the authors found significant reduction of the axial diffusivity and ADC in the corpus callosum in boys with the Tourette syndrome compared with the normal control. This may suggest significant changes in the white matter microstructure of the corpus callosum to contribute to the tic symptom itself, and other confounders like consequences of long-term medication, tic performance or suppression may not play a significant role.

DTI can be used to investigate white matter structures in patients and allow reconstruction of white matter tracts through estimation of the major fiber direction.^[27,28]^ Advancement in the DTI technique and tracking approaches have promoted the progress of neuronal connection models to quantify anatomical associations between different cerebral areas.^[29]^ With the DTI technique, distinctive changes between patients with Tourette syndrome and normal controls have been found in motor connections as well as in white matter tracts of fronto-striatal, somatosensory, and transcallosal circuits.^[19,21,22]^

Tic initiation has been associated with disturbed networks of cerebral regions in planning, adjusting and executing of actions, especially functional and structural diseases in the striatum and cortico–striato–thalamo–cortical loops.^[27]^ In a study applying the structural DTI to detect alterations in intrahemispheric white matter connection in the cortico-subcortical circuits for motor control in patients with Tourette syndrome without comorbidities of psychiatry,^[27]^ the white matter connection was assessed via probabilistic fiber tractography in 12 pre-defined cortical compared to the subcortical regions of interest. Widespread structural connectivity deficits were found in patients with the Tourette syndrome, and lower connection values were particularly detected in tracts linking the supplementary motor regions with basal ganglia (pre-supplementary motor area-putamen and supplementary motor area-putamen) and in frontal cortico-cortical circuits. An overall trend towards negative correlations was found between structural connection in these tracts and the Yale Global Tic Severity Scale scores. Structural connection of cerebral frontal networks engaged in planning, adjusting and executing actions is decreased in adult patients, and this connectivity is associated with the tic severity. Decreased connection values were revealed in almost two-thirds of all connections, which indicates a disruption in the neural networks involved in movement production and control. These findings may indicate the concept of the Tourette syndrome as a neurodevelopmental disease of cerebral immaturity.^[27]^ In our study, abnormality and damage to the morphology and course of the corticospinal tract, fiber bundles in the compression part of the corpus callosum, the inferior frontal occipital tract, and nerve fiber connection. This is a necessary addition to the current literature on the Tourette syndrome in children.

In a study investigating the structural abnormalities in Children of early Tourette syndrome,^[25]^ it was found that cerebral volume alterations were detected in the left superior temporal gyrus, right precuneus cortex, right and left paracentral gyrus, right pre- and postcentral gyrus, left lingual gyrus, left temporal occipital fusiform cortex, and right frontal pole. Great axial diffusivity and ADC elevations were found in the anterior thalamic radiation and right cingulum bundle projecting to the cingulate gurus and forceps minor. Drops in the white matter volume in the right frontal pole were in an inverse relation with tic severity, and rises in the axial diffusivity and ADC were in a positive correlation with tic severity and duration, respectively. These findings may suggest signs of neural plasticity corresponding to the experiential need.

## 5. Limitations

This study has some limitations, including the retrospective single-center study design, no randomization, a small sample of patients, and Chinese patients enrolled only, which may all affect the generalization of the outcome. Future prospective, multicenter, and controlled studies will have to be performed with involvement of multiple races and ethnicities and a relatively large sample of patients to achieve better outcomes.

## 6. Conclusion

In summary, further research is needed on the neural plasticity and functional compensation mechanism in children with Tourette syndrome, and the application of new MRI technologies has promoted the research and treatment of children with the Tourette syndrome. During MRI scanning, it is important to control head movements, and the accuracy of the study can also be improved by increasing the sample size. This study used the DTI technique and observed the integrity of the microstructure of the corticospinal tract, the compression part of the corpus callosum, and the inferior frontal occipital tract, providing imaging support for revealing the neuropathological mechanism of the Tourette syndrome in children.

## Author contributions

**Conceptualization:** Hong-Xin Jiang, Yan-Mei Ju, Shu-Man Han, Wen-Juan Wu.

**Data curation:** Hong-Xin Jiang, Yan-Mei Ju, Ting-Ting Gao, Jian-Li Sun, Hong-Gang Wang, Lei Cao, Shu-Man Han, Hui-Zhao Wu, Jin-Xu Wen, Bu-Lang Gao, Wen-Juan Wu.

**Formal analysis:** Hong-Xin Jiang, Yan-Mei Ju, Ting-Ting Gao, Lei Cao, Jin-Xu Wen, Wen-Juan Wu.

**Funding acquisition:** Bu-Lang Gao.

**Investigation:** Hong-Xin Jiang, Yan-Mei Ju, Ting-Ting Gao, Jian-Li Sun, Hong-Gang Wang, Lei Cao, Shu-Man Han, Hui-Zhao Wu, Jin-Xu Wen, Bu-Lang Gao, Wen-Juan Wu.

**Methodology:** Hong-Xin Jiang, Jian-Li Sun, Hong-Gang Wang, Hui-Zhao Wu.

**Project administration:** Hong-Xin Jiang, Yan-Mei Ju, Jian-Li Sun, Wen-Juan Wu.

**Resources:** Hong-Gang Wang, Lei Cao, Wen-Juan Wu.

**Software:** Hong-Xin Jiang.

**Supervision:** Hong-Xin Jiang, Yan-Mei Ju, Shu-Man Han, Hui-Zhao Wu, Jin-Xu Wen, Wen-Juan Wu.

**Validation:** Hong-Xin Jiang, Yan-Mei Ju, Ting-Ting Gao, Jian-Li Sun, Hong-Gang Wang, Lei Cao, Shu-Man Han, Hui-Zhao Wu, Jin-Xu Wen, Bu-Lang Gao, Wen-Juan Wu.

**Visualization:** Hong-Xin Jiang, Yan-Mei Ju, Ting-Ting Gao, Jian-Li Sun, Hong-Gang Wang, Lei Cao, Shu-Man Han, Hui-Zhao Wu, Jin-Xu Wen, Bu-Lang Gao, Wen-Juan Wu.

**Writing – original draft:** Hong-Xin Jiang.

**Writing – review & editing:** Bu-Lang Gao, Wen-Juan Wu.
